# Alterations in the insulin-like growth factor system during treatment with diethylstilboestrol in patients with metastatic breast cancer

**DOI:** 10.1054/bjoc.2001.1871

**Published:** 2001-07

**Authors:** S I Helle, J Geisler, G B Anker, B Leirvaag, J M P Holly, P E Lønning

**Affiliations:** Department of Oncology, Haukeland University Hospital, Bergen, N-5021, Norway; Division of Surgery, Bristol Royal Infirmary, Bristol, BS2 HW, UK

**Keywords:** high dose oestrogens, breast cancer therapy, IGF-system

## Abstract

Alterations in the insulin-like growth factor (IGF)-system were evaluated in 16 patients treated with diethylstilboestrol 5 mg 3 times daily. Fasting blood samples were obtained before treatment and after 2 weeks, 1 month and/or 2–3 months on therapy. Insulin-like growth factor (IGF)-I, IGF-II, free IGF-I, IGF-binding protein (IGFBP)-1, IGFBP-2 and IGFBP-3 were measured by radioimmuno-/immunoradiometric-assays. All samples were subjected to Western ligand blotting as well as immunoblotting for IGFBP-3. We observed a significant decrease (percentage of pretreatment levels with 95 confidence intervals of the mean) in IGF-I [2 weeks 63% (49–79); 1 month 56% (44–73); 2–3 months 66% (53–82)], IGF-II [2 weeks 67% (56–80); 1 month 60% (52–68); 2–3 months 64% (55–75)], free IGF-I [2 weeks 29% (19–42); 1 month 25% (18–36); 2–3 months 31% (21–46)], IGFBP-2 [2 weeks 53% (18–156); 1 month 69% (61–78); 2–3 months 66% (57–78)], IGFBP-3 [2 weeks 74% (63–85); 1 month 69% (62–76); 2–3 months 71% (63–80)], as well as IGFBP-3 protease activity [2 weeks 71% (54–95); 1 month 78% (64–94); 2–3 months 71% (54–93)]. Contrary, the plasma levels (percentage of pretreatment levels with 95 confidence intervals of the mean) of IGFBP-1 [2 weeks 250% (127–495); 1 month 173% (138–542); 2–3 months 273% (146–510)] and IGFBP-4 [2 weeks 146% (112–192); 1 month 140% (116–169); 2–3 months 150% (114–198)] increased significantly. While this study confirms previous observations during treatment with oral oestrogens in substitution doses, the reduction in plasma IGF-II, free IGF-I, IGFBP-2 and -3 are all novel findings. A profound decrease in free IGF-I suggests a reduced bioavailability of IGFs from plasma to the tissues. These observations may be of significance to understand the mechanisms of the antitumour effect of diethylstilboestrol in pharmacological doses. © 2001 Cancer Research Campaign http://www.bjcancer.com


*British Journal of Cancer* (2001) **85**(2), 147–151
© 2001 Cancer Research Campaign
doi: 10.1054/ bjoc.2001.1871, available online at http://www.idealibrary.com on IDEAL

http://www.bjcancer.com

### Alterations in the insulin-like growth factor system during treatment with diethylstilboestrol in patients with metastatic breast cancer

**SI Helle[1], J Geisler[1], GB Anker[1], B Leirvaag[1], JMP Holly[2] and PE Lønning[1]**

[1]Department of Oncology, Haukeland University Hospital, N-5021 Bergen, Norway and [2]Division of Surgery, Bristol Royal Infirmary, BS2 HW Bristol, UK

**Summary** Alterations in the insulin-like growth factor (IGF)-system were evaluated in 16 patients treated with diethylstilboestrol 5 mg 3 times daily. Fasting blood samples were obtained before treatment and after 2 weeks, 1 month and/or 2–3 months on therapy. Insulin-like growth factor (IGF)-I, IGF-II, free IGF-I, IGF-binding protein (IGFBP)-1, IGFBP-2 and IGFBP-3 were measured by radioimmuno-/immunoradiometric-assays. All samples were subjected to Western ligand blotting as well as immunoblotting for IGFBP-3. We observed a significant decrease (percentage of pretreatment levels with 95 confidence intervals of the mean) in IGF-I [2 weeks 63% (49–79); 1 month 56% (44–73); 2–3 months 66% (53–82)], IGF-II [2 weeks 67% (56–80); 1 month 60% (52–68); 2–3 months 64% (55–75)], free IGF-I [2 weeks 29% (19–42); 1 month 25% (18–36); 2–3 months 31% (21–46)], IGFBP-2 [2 weeks 53% (18–156); 1 month 69% (61–78); 2–3 months 66% (57–78)], IGFBP-3 [2 weeks 74% (63–85); 1 month 69% (62–76); 2–3 months 71% (63–80)], as well as IGFBP-3 protease activity [2 weeks 71% (54–95); 1 month 78% (64–94); 2–3 months 71% (54–93)]. Contrary, the plasma levels (percentage of pretreatment levels with 95 confidence intervals of the mean) of IGFBP-1 [2 weeks 250% (127–495); 1 month 173% (138–542); 2–3 months 273% (146–510)] and IGFBP-4 [2 weeks 146% (112–192); 1 month 140% (116–169); 2–3 months 150% (114–198)] increased significantly. While this study confirms previous observations during treatment with oral oestrogens in substitution doses, the reduction in plasma IGF-II, free IGF-I, IGFBP-2 and -3 are all novel findings. A profound decrease in free IGF-I suggests a reduced bioavailability of IGFs from plasma to the tissues. These observations may be of significance to understand the mechanisms of the antitumour effect of diethylstilboestrol in pharmacological doses. © 2001 Cancer Research Campaign http://www.bjcancer.com

**Keywords**: high dose oestrogens; breast cancer therapy; IGF-system

Endocrine therapy has a key role in the treatment of metastatic breast cancer. While oestrogens given in pharmacological doses have been known for decades to cause antitumour effects (Haddow et al, 1944; Carter et al, 1977), they were replaced by tamoxifen in the late 1970s. The reason to this was the lower toxicity of the antioestrogen, while the 2 regimens were considered to be equally effective with respect to tumour response (Ingle et al, 1981). Long-term follow-up results have suggested diethylstilboestrol to be more effective than tamoxifen (Peethambaram et al, 1999).

The mechanisms by which oestrogens in high doses cause anti-tumour effects in breast cancer are not known. However, in vitro studies have focused on the importance of cross-talk between steroids and growth factors, in particular the insulin like growth factors (IGFs), to breast cancer growth (Westley and May, 1994; Lee et al, 1997).

IGF-I is a potent mitogen to breast cancer cell lines in vitro (Karey and Sirbasku, 1988) and high plasma levels of IGF-I has been reported to be a risk factor for breast cancer development in premenopausal women (Hankinson et al, 1998). Inhibition of IGF-I receptor activation by antibodies or antisense strategies has been shown to cause antitumour effects in animal models (Arteaga et al, 1989; Dunn et al, 1998). IGF-I and -II are expressed by many different tissues and may thus act by both paracrine and autocrine mechanisms in addition to the large circulating plasma pool of peptides (Daughaday and Rotwein, 1989). In addition, the bioavailability of IGF-I as well as IGF-II to the tissues may be influenced by plasma levels of the 6 different IGF-binding proteins (IGFBPs), IGFBP related proteins, phosphorylation status of the IGFBPs as well as IGFBP-protease activity (Jones and Clemmons, 1995; Hwa et al, 1998). About 99% of IGF-I and IGF-II are found in a ternary complex consisting of IGF-I or -II, IGFBP-3 (the major IGFBP in plasma) and an acid labile subunit (ALS). Thus, only a minor fraction of circulating IGFs is free (or easily dissociable) and therefore available to the tissues. Release of IGF-I from the circulating pool may be facilitated through increased activity of IGFBP-3 proteases, reducing the binding affinity of IGF-I to the ternary complex (Lassarre and Binoux, 1994). Increased IGFBP-3 protease activity is observed in several clinical conditions including advanced breast cancer (Giudice et al, 1990; Müller et al, 1993; Bereket et al, 1995; Cotterill et al, 1996; Frost et al, 1996).

Previous studies have revealed oral administration of oestrogens for hormone substitution (Weissberger et al, 1991; Helle et al, 1996c) as well as the SERMS tamoxifen and droloxifene (Colletti et al, 1989; Lønning et al, 1992; Helle et al, 1996a) to cause a moderate reduction in total IGF-I but elevated IGFBP-I. However, the effect of oestrogens in pharmacological doses on the IGF-system has not been reported previously.

To evaluate potential alterations in the IGF-system in breast cancer patients receiving oestrogens in pharmacological doses, we

*Received 25 September 2000*
*Revised 14 March 2001*
*Accepted 27 March 2001*

*Correspondence to:* PE Lønning measured IGF-I and IGF-II together with the IGF-binding proteins and IGFBP-3 protease activity in 16 patients suffering from metastatic breast cancer before and during treatment with diethylstilboestrol 5 mg 3 times daily.

### SUBJECTS AND METHODS

### Patients

16 postmenopausal women with progressive metastatic breast cancer failing multiple endocrine regimens were enrolled in a phase II study evaluating treatment with diethylstilboestrol 5 mg 3 times daily. The median age was 72 years (range 52–87), and the number of previous endocrine regimens ranged from 3 to 10 (median 4). Last treatment before diethylstilboestrol therapy and the length of the washout period (time between last dose of their previous therapy and pre-treatment blood sampling) is given in Table 1. The clinical results are to be reported elsewhere as a 2-centre study (Lønning et al, 2001). The study was approved by the regional ethical committee.

### Blood sampling

Fasting blood samples (heparinized vials) were obtained before treatment and after 2 weeks, 1 month and/or 2–3 months during treatment with diethylstilboestrol. Samples were centrifugated within 20 minutes and plasma aliquots stored at –20 C until analysis.

### Materials

Human recombinant IGF-I and IGF-II were purchased from GroPep (Adelaide, Australia). IGF-I and IGF-II were iodinated using the chloramine-T method. Labelled peptide was separated from non-incorporated $^{125}$I by AcA 202 columns (BioSepra, Villeneuve, France) using 1 × 40 cm columns.

### Assays

Plasma levels of IGF-I and IGF-II (Frost et al, 1996) were measured by RIA following acid-acetone extraction (Bowsher et al, 1991). Intra-and inter-assay coefficients of variations were 3.5% and 6.2% for IGF-I and 5.5% and 12.9% for IGF-II, respectively. Commercial kits (IRMA/RIA) for free IGF-I, IGFBP-I, IGFBP-2, and IGFBP-3 were purchased from Diagnostic System Laboratories (Webster, TX), and the measurements were made according to the manufacturer's instructions.

The IGFBP profile in the plasma was analysed by Western ligand blotting (WLB) using a modified version (Coulson et al, 1991) of the technique originally developed by Hossenlopp (Hossenlopp et al, 1986). $^{125}$I labelled IGF-I and IGF-II was used as tracer binding IGF-binding proteins on the membrane. Radiolabelled IGFBPs were visualized by autoradiography and quantified using a densitometric scanner (Pharmacia LKB, Uppsala, Sweden). The IGFBP pattern was compared with the profile of a normal plasma pool (NP), and samples from each patient were analysed in the same run for comparison. In order to analyse for IGFBP-3 proteolysis, the membranes were immunoblotted (after WLB) using a polyclonal antiserum against IGFBP-3 purchased from Diagnostic Systems Laboratories (Webster, TX) at a final dilution of 1:10 000. The membranes were then developed (showing intact IGFBP-3 as well as fragments) using enhanced chemiluminescent reagents supplied by Amersham (Aylesbury, UK) according to the manufacturer's instruction, and the films subjected to densitometric scanning. IGFBP-3 protease activity was measured indirectly as IGFBP-3 fragmentation, defined as the ratio of the major IGFBP-3 fragment (30kDa) to total IGFBP-3 evaluated by densitometric scanning of immunoblots.

### Statistics

Testing for distribution of the different IGF parameters with Q–Q plots in our normal populations of pre- and postmenopausal women revealed all parameters to be best fitted to a log normal distribution with the exception of IGFBP-3 protease activity. This parameter was best described by a normal distribution. Thus,

**Table 1** Demographic and clinical data of patients included in the study

| Patient no. | Age (years) | No. of previous endocrine regimens | Last treatment before DES | Response to DES | Washout period (days) |
|---|---|---|---|---|---|
| 1 | 86 | 3 | Formestane | CR | 30 |
| 2 | 53 | 6 | Formestane | PD | 8 |
| 3 | 65 | 3 | Tamoxifen | PR | 47 |
| 4 | 73 | 4 | Exemestane | PD | 14 |
| 5 | 74 | 5 | Anastrozole | PR | 14 |
| 6 | 76 | 5 | Tamoxifen | SD | 27 |
| 7 | 71 | 4 | Anastrozole | PD | 30 |
| 8 | 70 | 10 | Anastrozole | SD | 21 |
| 9 | 62 | 3 | MA | PR | 21 |
| 10 | 70 | 5 | Formestane | PD | 24 |
| 11 | 79 | 3 | MA + Formestane | CR | 14 |
| 12 | 87 | 5 | Tamoxifen | PR | 42 |
| 13 | 80 | 5 | Tamoxifen | CR | 791 |
| 14 | 72 | 5 | Tamoxifen | SD | 19 |
| 15 | 72 | 3 | MA | CR | 30 |
| 16 | 68 | 3 | Aminoglutethimide | PR | 36 |

Response was evaluated according to the UICC criteria; CR = complete response, PR = partial response, SD = stable disease, > 3 months, PD = progressive disease. MA = megestrol acetate. Washout period = time period from stopping last endocrine regimen to commencement of diethylstilbestrol.

 © 2001 Cancer Research Campaign

**Table 2** Values of IGF-I, F-IGF-I, IGF-II, IGFBP-1, IGFBP-2 and IGFBP-3 measured by RIA/IRMA, and IGFBP-2, -3 and -4 by Western ligand blot before treatment, and percentage of pretreatment levels/percentage change at various intervals during treatment with diethylstilboestrol. IGFBP-3 protease activity is measured indirectly as the ratio of fragmented to total IGFBP-3 on immunoblots. Values are given as geometrical mean values (with 95% confidence intervals) with the exception of IGFBP-3 protease where arithmetic values are given. During treatment alterations in IGFBP-3 protease activity are given as percentage change, while the other parameters are given as percentage of pretreatment values

| | Measured levels (nmol $l^{-1}$) percentage change/percentage of pretreatment levels | | | |
|---|---|---|---|---|
| | **Before treatment (*n* = 16)** | **2 weeks (*n* = 12)** | **1 month (*n* = 14)** | **2–3 months (*n* = 14)** |
| IGF-I | 12.8 (10.6–15.5) | 63 (49–79) | 56 (44–73) | 66 (53–82) |
| IGF-II | 64.4 (51.3–80.9) | 67 (56–80) | 60 (52–68) | 64 (55–75) |
| F-IGF-I | 0.10 (0.08–0.14) | 29 (19–42) | 25 (18–36) | 31 (21–46) |
| IGFBP-1 RIA | 1.5 (0.7–3.0) | 250 (127–495) | 273 (138–542) | 273 (146–510) |
| IGFBP-2 RIA | 29.6 (23.1–38.0) | 53 (18–156) | 69 (61–78) | 66 (57–78) |
| IGFBP-2* WLB | 5.1 (2.9–8.8) | 80 (65–99) | 68 (52–88) | 57 (44–74) |
| IGFBP-3 RIA | 94.6 (82.9–106.3) | 74 (63–85) | 69 (62–76) | 71 (63–80) |
| IGFBP-3* WLB | 75 (57–97) | 84 (64–110) | 61 (45–82) | 69 (52–92) |
| IGFBP-3** PROT | 0.35 (0.30–0.42) | –29 (–46 –5)*** | –22 (–36 –6)*** | –29 (–46 –7)*** |
| IGFBP-4* WLB | 3.7 (2.7–5.1) | 146 (112–192) | 140 (116–169) | 150 (114–198) |

*Arbitrary units. **Ratio of fragmented to total IGFBP-3. *n* = number of observations.
*** Percentage change from pretreatment levels.

parameters obtained in the different groups of patients are given as their geometric mean value with 95% confidence intervals of the mean, with the exception of IGFBP-3 protease activity where the arithmetic mean values are given. Considering statistical difference from pre-treatment levels, on-treatment values are significantly different when the confidence interval of the mean does not span the mean pretreatment value. *P* values were calculated using the Friedmans test (non-parametric analysis of variance).

### RESULTS

9 patients obtained a partial or a complete response to treatment, 3 stable disease, while progressive disease was observed in 4 patients. Median time to progression was 18 weeks.

Alterations in the different IGF-parameters during treatment with diethylstilboestrol are shown in Table 2. The findings may be summarized as follows.

Treatment with diethylstilboestrol suppressed plasma IGF-I to a mean value of 56–66% of pretreatment levels ($P < 0.001$), IGF-II to 60–67% of pretreatment levels ($P < 0.001$) and free IGF-I to 25–31% of its pretreatment values ($P < 0.001$). Similarly, we observed a decrease in plasma IGFBP-2 to 53–69% of its pretreatment values as measured by RIA ($P = 0.001$), and 57–80% of pretreatment values when evaluated by Western ligand blot ($P = 0.0025$). We also observed a decrease in IGFBP-3 measured by RIA (69–74% of pretreatment values; $P = 0.001$), and Western ligand blot (61–84% of pretreatment values; $P = 0.07$), as well as a decrease in IGFBP-3 protease activity (mean decrease of 22–29%; $P = 0.03$). In contrast, plasma levels of IGFBP-1 measured by RIA and IGFBP-4 measured by Western ligand blot both increased (IGFBP-1 to 250–273% of pretreatment values; $P = 0.001$, and IGFBP-4 to 140–150% of pretreatment values; $P = 0.03$).

Previous treatment could potentially affect the results for patients number 2, 6 and 14. However, eliminating these patients had no influence on the results with the exception of IGFBP-3 protease activity, where the significant decrease in this parameter was lost.

Representative Western ligand blots and immunoblots are shown in Figure 1. For most parameters these alterations were fully developed after 2 weeks on treatment. Comparing patients obtaining a clinical response (CR or PR), stable disease, and progressive disease did not reveal any significant differences in the measured parameters between these patient groups.

### DISCUSSION

Plasma levels of the different IGF-parameters measured before treatment were in the same range as previously reported by our group (Frost et al, 1996). Although some patients had short washout time, it is unlikely that previous treatment has significant impact on our results as most of these patients had received formestane, exemestane or anastrozole. Formestane (Frost et al, 1996) and exemestane (unpublished observations by our group)

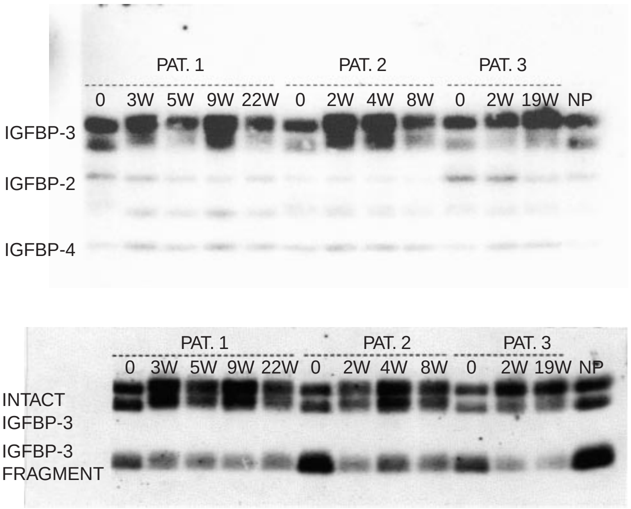

**Figure 1** Western ligand blots (**A**) with corresponding immunoblots (**B**) from 3 patients before and during treatment with diethylstilboestrol. As these patients had a decrease in IGFBP-3 protease activity during treatment, alterations in intact IGFBP-3 do not reflect the fall in total 16FBP-3 observed in the whole treatment group

© 2001 Cancer Research Campaign have been shown to have little effects on plasma IGF-I, while no data exists on anastrozole.

The moderate reduction in total IGF-I (30–40%) found in this study is in the range previously reported for oral oestrogens administered in doses regularly used for hormone replacement (Weissberger et al, 1991; Helle et al, 1996c), and during treatment with tamoxifen (Colletti et al, 1989; Helle et al, 1996b) or droloxifene (Helle et al, 1996a) in breast cancer patients. While these drugs also cause an increase in IGFBP-1 (Lønning et al, 1992; Helle et al, 1996a), the magnitude of this increase seems smaller with droloxifene, tamoxifen and hormone replacement therapy compared to that which we describe here. This may be related to drug doses for tamoxifen and droloxifene but also to the relative oestrogen agonistic versus antagonistic effects of these drugs. Tamoxifen has oestrogen agonistic effects on hepatic synthesis of sex hormone-binding globuline, thyroxin-binding globuline as well as lipoproteins (Fex et al, 1981; Love et al, 1991) and this drug is found to inhibit IGF-I gene expression in the liver (Huynh et al, 1993). Thus it is likely that the effects on plasma IGF-I levels (and possibly some IGFBPs) are due to oestrogen agonistic effects of diethylstilboestrol on hepatic synthesis.

Findings from in vitro studies are of limited value to explain the effects of diethylstilboestrol on plasma IGF-II and IGF-binding proteins. Oestradiol has been reported to increase expression of IGF-II (Lee et al, 1994), IGFBP-2 (Pratt and Pollak, 1993) and enhance the secretion of IGFBP-3 proteases (Salahifar et al, 2000) in breast cancer cell lines, while the opposite effects on plasma levels of these parameters are observed in this study.

The significant reduction in plasma IGF-II (30–40%) observed here, has not been recorded previously during therapy with oestrogens or anti-oestrogens. In contrast to IGF-I, IGF-II has not been considered a subject to regulation by endocrine drugs. The decrease in IGF-II observed in this study may be secondary to a decrease in IGFBP-3, which is also observed during treatment with oral oestrogens in replacement doses (Kam et al, 2000) as well as somatostatin analogues (Helle et al, 1998). However, in contrast to somatostatin analogues which decrease plasma growth hormone (GH) levels, the opposite effect is found during treatment with diethylstilboestrol (Bishop et al, 1985).

To our knowledge, free IGF-I has not been measured in previous studies evaluating the effect of endocrine treatment on the IGF-system. Here, we observed a drop in plasma levels of free IGF-I to a mean value of 25–31% of its pretreatment level. The decrease in free IGF-I may be somewhat more pronounced than expected based on the moderate decrease in total IGF-I. The observed decrease in IGFBP-3 protease activity may to some extent explain this finding, as the IGFBP-3 protease may facilitate release of IGF-I from the ternary complex (Lassarre and Binoux, 1994). The commercial IRMA kit used for measurement of free IGF-I in this study in general measures somewhat higher levels compared to what is recorded with use of ultrafiltration, probably due to inclusion of some easily dissociable IGF-I (Frystyk et al, 1999). However, the value most likely reflects the amount of IGF-I readily available to the tissues. It is also possible that increase in plasma levels of IGFBP-1 and IGFBP-4 may contribute to the reduced levels of free IGF-I. A greater suppression of free IGF-I, compared to total IGF-I, may indicate that free IGF-I measurement has more value as a surrogate marker for IGF-I bioavailability for breast cancer patients treated with endocrine therapy.

A moderate reduction in IGFBP-3 protease was observed. The patients included in the study in general had a low metastatic tumour burden and a slowly progressive disease. A high number of our patients responded to therapy with diethylstilboestrol, and we have recorded a decrease in IGFBP-3 protease activity for patients responding to tamoxifen (Helle et al, 1996b). A decrease in IGFBP-2 was also observed. Increased IGFBP-2 levels have been previously described for patients with prostate cancer (Cohen et al, 1993; Kanety et al, 1993). The mechanism behind the alterations in IGFBP-2 is not known, but there seems to be a strong positive correlation between IGFBP-3 protease activity and IGFBP-2 levels (unpublished observations). Thus, a decrease in IGFBP-2 may partly reflect a decrease in tumour burden during therapy.

The mechanisms by which oestrogens in high doses cause anti-tumour effects in breast cancer is unknown, but in vitro studies have shown a biphasic response curve for breast cancer cells with oestrogens in high concentrations being toxic to tumour cell growth (Lippman et al, 1976; Masamura et al, 1995). Also, there is evidence that oestrogens in high concentrations may induce apoptosis in vivo (Song et al, 2000). The toxicity threshold is lowered as an adaptive process during oestrogen deprivation (Masamura et al, 1995). Whether components of the IGF-system may be involved in these mechanisms is not known. The induction of the IGF-I receptor as well as the insulin receptor substrate-1 by oestrogens in breast cancer cell lines may indicate synergistic effects between these systems. (Stewart et al, 1990; Molloy et al, 2000). There is also evidence that IGF-I signalling may be necessary for maximal oestrogen receptor activation in some human breast cancer cell lines (Lee et al, 1997). The profound decrease in free IGF-I, and decrease in both IGF-I and -II, observed in this study, may be a part of the mechanism of action of this drug in breast cancer. However, further studies are needed to evaluate the complex interactions between oestrogens and the IGF-system in human breast cancer.

### ACKNOWLEDGEMENTS

This work was supported by the Norwegian Cancer Society. The technical assistance of Mr Dagfinn Ekse and Mrs Hildegunn Helle is highly appreciated.

### REFERENCES

Arteaga CJ, Kitten LJ, Coronado EB, Jacobs S Jr FCK Allred DC and Osborne CK (1989) Blockade of the type I somatomedin receptor inhibits growth of human breast cancer cells in athymic mice. *J Clin Invest* **84**: 1418–1423

Bereket A, Lang CH, Blethen SL, Fan J, Frost RA and Wilson TA (1995) Insulin-like growth factor binding protein-3 proteolysis in children with insulin-dependent diabetes mellitus: a possible role for insulin in the regulation of IGFBP-3 protease activity. *J Clin Endocrinol Metab* **80**: 2282–2288

Bishop MC, Selby C and Taylor M (1985) Plasma hormone levels in patients with prostatic carcinoma treated with diethylstilboestrol and estramustine. *Br J Urol* **57**: 542–547

Bowsher RR, Lee W-H, Apathy JM, O'Brien PJ, Ferguson AL and Henry DP (1991) Measurement of insulin-like growth factor-II in physiological fluids and tissues. I. An improved extraction procedure and radioimmunoassay for human and rat fluids. *Endocrinology* **128**: 805–814

Carter AC, Sedransk N, Kelley RM, Ansfield FJ, Ravdin RG, Talley RW and Potter NR (1977) Diethylstilbestrol: Recommended dosages for different categories of breast cancer patients. *JAMA* **237**: 2079–2085

Cohen P, Peehl DM, Stamey TA, Wilson KF, Clemmons DR and Rosenfeld RG (1993) Elevated levels of insulin-like growth factor-binding protein-2 in the serum of prostate cancer patients. *J Clin Endocrinol Metab* **76**: 1031–1035

Colletti RB, Roberts JD, Devlin JT and Copeland KC (1989) Effect of tamoxifen on plasma insulin-like growth factor I in patients with breast cancer. *Cancer Res* **49**: 1882–1884

 *© 2001 Cancer Research Campaign*

Cotterill AM, Mendel P, Holly JMP, Timmins AG, Camacho-Hubner C, Cwyfan-Hughes S, Ross RMJ, Blum WF and Langford RM (1996) The differential regulation of the circulating levels of the insulin-like growth factors and their binding proteins (IGFBP) 1,2 and 3 after elective abdominal surgery. *Clin Endocrinol* **44**: 91–101

Coulson VJ, Wass JAH, Abdulla AF, Cotterill AM and Holly JMP (1991) Insulin-like growth factor binding proteins (IGFBPs) in acromegaly. *Growth Reg* **1**: 119–124

Daughaday WH and Rotwein P (1989) Insulin-like growth factors I and II. Peptide messinger ribonucleic acid and gene structures, serum, and tissue concentrations. *Endocrine Rev* **10**: 68–91

Dunn SE, Ehrlich M, Sharp NJH, Reiss K, Solomon G, Hawkins R, Baserga R and Barrett JC (1998) A dominant negative mutant of the insulin-like growth factor-I receptor inhibits the adhesion, invasion, and metastasis of breast cancer. *Cancer Res* **58**: 3353–3361

Fex G, Adielsson G and Mattson W (1981) Oestrogen-like effects of tamoxifen on the concentration of proteins in plasma. *Acta Endocrinol* **97**: 109–113

Frost VJ, Helle SI, Lønning PE, van der Stappen JWJ and Holly JMP (1996) Effects of treatment with megestrol acetate, aminoglutethimide or formestane on insulin-like growth factor (IGF) I and II, IGF-binding proteins (IGFBPs) and IGFBP-3 protease status in patients with advanced breast cancer. *J Clin Endocrinol Metab* **81**: 2216–2221

Frystyk J, Skjærbæk C, Støvning RK, Dall R, Bek T and Ørskov H (1999) Measurement of free insulin-like growth factor-I in human serum: comparison of ultrafiltration and direct immunoradiometric assay. *Growth Horm IGF Res* **9**: P45

Giudice LC, Farrell EM, Pham H, Lamson G and Rosenfeld RG (1990) Insulin-like growth factor binding proteins in maternal serum throughout gestation and in the puerperium: effects of a pregnancy associated serum protease activity. *J Clin Endocrinol Metab* **71**: 806–816

Haddow A, Watkinson JM and Paterson E (1944) Influence of synthetic oestrogens upon advanced malignant disease. *Br Med J* **2**: 393–398

Hankinson SE, Willett WE, Colditz GA, Hunter DJ, Michaud DS, Deroo B, Rosner B, Speizer FE and Pollak M (1998) Circulating concentrations of insulin-like growth factor-I and risk of breast cancer. *Lancet* **351**: 1393–1396

Helle SI, Anker GB, Tally M, Hall K and Lønning PE (1996a) Influence of droloxifene on plasma levels of insulin-like growth factor (IGF)-I, pro-IGF-IIE, insulin-like growth factor binding protein (IGFBP)-1 and IGFBP-3 in breast cancer patients. *J Steroid Biochem Molec Biol* **57**: 167–171

Helle SI, Holly JMP, Tally M, Hall K, van der Stappen J and Lønning PE (1996b) Influence of treatment with tamoxifen and change in tumour burden on the IGF-system in breast cancer patients. *Int J Cancer* **69**: 335–339

Helle SI, Omsjø IH, Cwyfan-Hughes SC, Holly JMP and Lønning PE (1996c) Effects of oral and parenteral oestrogen replacement therapy on plasma levels of insulin-like growth factors (IGFs) and IGF binding proteins 1 and 3: a crossover study. *Clin Endocrinol* **45**: 727–732

Helle SI, Geisler J, Poulsen JP, Hestdal K, Meadows K, Collins W, Tveit KM, Viste A, Holly JMP and Lønning PE (1998) Microencapsulated octreotide pamoate in advanced gastrointestinal and pancreatic cancer: a phase I study. *Br J Cancer* **78**: 14–20

Hossenlopp P, Seurin D, Segovia-Quinson B, Lassarre C, Hardouin S and Binoux M (1986) Analysis of serum insulin-like growth factor binding proteins using Western ligand blotting: use of the method of titration of the binding proteins in competitive binding studies. *Anal Biochem* **154**: 138–143

Huynh HT, Tetenes E, Wallace L and Pollak M (1993) *In vivo* inhibition of insulin-like growth factor I gene expression by tamoxifen. *Cancer Res* **53**: 1727–1730

Hwa V, Tomasini-Sprenger C, Bermejo AL, Rosenfeld RG and Plymate SR (1998) Characterization of insulin-like growth factor-binding protein-related protein-1 in prostate cells. *J Clin Endocrinol Metab* **83**: 4355–4362

Ingle JN, Ahmann DL, Green SJ, Edmonson JH, Bisel HF, Kvols LK, Nichols WC, Creagan ET, Hahn RG, Rubin J and Frytak S (1981) Randomized clinical trial of diethylstilstilbestrol versus tamoxifen in postmenopausal women with advanced breast cancer. *N Engl J Med* **304**: 16–21

Jones JI and Clemmons DR (1995) Insulin-like growth factors and their binding proteins: biological actions. *Endocrine Rev* **16**: 3–34

Kam GYW, Leung K-C, Baxter RC and Ho KKY (2000) Estrogens exert route-and dose-dependent effects on insulin-like growth factor (IGF)-binding protein-3 and the acid-labile subunit of the IGF ternary complex. *J Clin Endocrinol Metab* **85**: 1918–1922

Kanety H, Madjar Y, Dagan Y, Levi J, Papa MZ, Pariente C, Goldwasser B and Karasik A (1993) Serum insulin-like growth factor-binding protein-2 (IGFBP-2) is increased and IGFBP-3 is decreased in patients with prostate cancer: correlation with serum prostate-specific antigen. *J Clin Endocrinol Metab* **77**: 229–233

Karey KP and Sirbasku DA (1988) Differential responsiveness of human breast cancer cell lines MCF-7 and T47D to growth factors and 17β-estradiol. *Cancer Res* **48**: 4083–4092

Lassarre C and Binoux M (1994) Insulin-like growth factor binding protein-3 is functionally altered in pregnancy plasma. *Endocrinology* **134**: 1254–1262

Lee AV, Darbre P and King RJB (1994) Processing of insulin-like growth factor-II (IGF-II) by human breast cancer cells. *Mol Cell Endocrinol* **99**: 211–220

Lee AV, Weng C-N, Jackson JG and Yee D (1997) Activation of estrogen receptor-mediated gene transcription by IGF-I in human breast cancer cells. *J Endocrinol* **152**: 39–47

Lippman M, Bolan G and Huff K (1976) The effects of estrogens and antiestrogens on hormone-responsive human breast cancer in long-term tissue culture. *Cancer Res* **36**: 4595–4601

Lønning PE, Hall K, Aakvaag A and Lien EA (1992) Influence of tamoxifen on plasma levels of insulin-like growth factor I and insulin-like growth factor binding protein 1 in breast cancer patients. *Cancer Res* **52**: 4719–4723

Lønning PE, Taylor PD, Anker G, Iddon J, Wie L, Jørgensen L-M, Mella O and Howell A (2001) High-dose estrogen treatment in postmenopausal breast cancer patients heavily exposed to endocrine therapy. *Breast Cancer Res Treat* in press

Love RR, Wiebe DA, Newcombe PA and al., e. (1991) Effects of tamoxifen on cardiovascular risk factors in postmenopausal women. *Ann Intern Med* **115**: 860–864

Masamura S, Santner SJ, Heitjan DF and Santen RJ (1995) Estrogen deprivation causes estradiol hypersensitivity in human breast cancer cells. *J Clin Endocrinol Metab* **80**: 2918–2925

Molloy CA, May FEB and Westley BR (2000) Insulin receptor substrate-1 expression is regulated by estrogen in the MCF-7 human breast cancer cell line. *J Biol Chem* **275**: 12565–12571

Müller HL, Oh Y, Gargosky SE, Lehrnbecher T, Hinz RL and Rosenfeld RG (1993) Concentrations of insulin-like growth factor (IGF)-binding protein-3 (IGFBP-3), IGF, and IGFBP-3 protease activity in cerebrospinal fluid of children with leukemia, central nervous system tumor, or meningitis. *J Clin Endocrinol Metab* **77**: 1113–1119

Peethambaram PP, Ingle JN, Suman VJ, Hartmann LC and Loprinzi CL (1999) Randomized trial of diethylstilbestrol vs. tamoxifen in postmenopausal women with metastatic breast cancer. An updated analysis. *Breast Cancer Res Treat* **54**: 117–122

Pratt SE and Pollak MN (1993) Estrogen and antiestrogen modulation of MCF7 human breast cancer cell proliferation is associated with specific alterations in accumulation of insulin-like growth factor-binding proteins in conditioned media. *Cancer Res* **53**: 5193–5198

Salahifar H, Baxter RC and Martin JL (2000) Differential regulation of insulin-like growth factor-binding protein-3 protease activity in MCF-7 breast cancer cells by estrogen and transforming growth factor-betal. *Endocrinology* **141**: 3104–3110

Song RX, McPherson R, Yue W, Wang JP and Santen RJ (2000) Estrogen induces apoptosis in human breast cancer cells adapted to long term estrogen deprivation. *Proc Am Assoc Cancer Res* **41**: 2715

Stewart AJ, Johnson MD, May FEB and Westley BR (1990) Role of the insulin-like growth factors and the type I insulin-like growth factor receptor in the estrogen-stimulated proliferation of human breast cancer cells. *J Biol Chem* **265**: 21172–21178

Weissberger AJ, Ho KKY and Lazarus L (1991) Contrasting effects of oral and transdermal routes of estrogen replacement on 24 hour growth hormone (GH) secretion, insulin-like growth factor-I, and GH-binding protein in postmenopausal women. *J Clin Endocrinol Metab* **72**: 374–381

Westley BR and May FEB (1994) Role of the insulin-like growth factors in steroid modulated proliferation. *J Steroid Bioch, Molec Biol* **51**: 1–9

© 2001 Cancer Research Campaign 
